# The Death of Mr. Ernest Hart

**Published:** 1898-01-15

**Authors:** 


### THE DEATH OF MR. ERNEST HART.

WE regret to have to record the death of Mr. Ernest Hart, which took place at Brighton on Friday morning, January 7th. He had long been an invalid, having been affected in varying degree with diabetes for many years. During the past year he had also suffered much pain in the leg, where necrotic changes ultimately developed rendering amputation necessary. After the operation considerable relief was experienced, although he continued to suffer much from pain. Still, he was able to be removed to Brighton, and hope was felt that he might be restored to a certain amount of health. These hopes were, however, to be disappointed, and he died, as we have said, last Friday.

Mr. Hart was born in London in 1836, and received his early education at the City of London School. He studied medicine at St. George's Hospital, and became a member of the Royal College of Surgeons in 1856. On commencing practice he devoted himself much to ophthalmic work, and, at the age of 28, was appointed ophthalmic surgeon at St. Mary's Hospital, where he became dean of the medical school.

It was, however, in journalistic work that Mr. Hart found the most congenial field for his wide sympathies, and in which he was able to put his versatile powers to their best use, and his death removes from the field of medical journalism a figure which for the past thirty years has been constantly and prominently before the public eye. At an early age he became connected with the *Lancet*, and while there he took a leading part in organising those investigations into the conditions of the London workhouses which resulted in the appointment of a commission of inquiry, and ultimately in the passing of the Metropolitan Poor Act, under which separate infirmaries were established, sick asylum districts were arranged, and the Metropolitan Asylums Board was brought into being.

In 1866, that is, when 30 years of age, he was appointed to the editorship of the *British Medical Journal*, an appointment which he held until his death, and it has been as editor of the *British Medical Journal* that Mr. Hart has been most widely known, and that his greatest journalistic successes have been attained.

When he took office both the association and its journal were very small affairs in comparison with the position which they now occupy, and there is but little doubt that both owe a great deal of their prosperity to the energy which Mr. Hart imported into the conduct of the journal. The building up of so great an association was a matter of which any man might be proud, and Mr. Hart was never tired of maintaining that it was by the aid of the journal that the association was led on to influence and power. As everyone is aware, there always have been some who have doubted this, and have protested against the methods of the *British Medical Journal*. But those methods spelt success; and, much as the purists might protest, events have shown that Mr. Hart was right. He had the wit to see that if the journal was to have influence it must not address itself to the medical profession alone, but must do as the *Lancet* had done in its early days—must contrive to make itself heard by the outside world. It has been as a journal treating of the events of the day, more especially from their medical aspect, that the *British Medical Journal* has been so prosperous; and this prosperity has hinged on the fact, at which many of his readers squirmed, that its editor was a many-sided man, with wide sympathies, and with an accurate perception of what the public wanted. This was gall and wormwood to many, but it was journalism, and it gave influence and power to an association which otherwise might easily have fizzled out. Yet even now there are men who profess to disbelieve the obligation the association is under to Mr. Hart.

The variety of his interests may be to some extent judged by his publications, which include the following: "Hospitals of the State," "Reports on Workhouse Infirmaries," "The Influence of Milk in Spreading Zymotic Disease," "The Mosaic Code of Sanitation," "Inquiry into the Practice of Baby Farming," "Regulation of Plumbers," "Smoke Abatement," "Local Government as It Is and as It Ought to Be," "The Ancient Art and Artists of Japan," "Hypnotism, Mesmerism, and the New Witchcraft," "The Truth about Vaccination," "The Sanitary Needs of India," and "The Sick Poor in Workhouses."

Among the most recent literary work which he undertook was the editing of the Masters of Medicine series of medical biographies, in which he was assisted by Mr. Louis Taylor, and the contribution of an article on the Etiology of Cholera to "Allbutt's System of Medicine," and one on Water-borne Diseases to the "Twentieth Century Practice of Medicine," both of which were prepared in conjunction with Dr. Solomon Smith. Mr. Hart also contributed articles at various times to the *Nineteenth Century*, the *Century*, the *Forum*, and other periodicals, and at one time he was editor of the *Sanitary Record*.

Much of Mr. Hart's most influential work was done on the Parliamentary Bills Committee of the British Medical Association, of which he was chairman for many years. He was also chairman of the National Health Society. He was a man who had a few enemies—bitter and vindictive; but he was one who also had many friends. His pen could indeed be savage, and when his indignation was aroused by some abuse he could say hard things, and take an artistic pleasure in the expression of his anger in the most pungent words at his disposal. But there was another side; and those who knew him and remember his courtesy and kindness, his wide love of humanity, his sympathy for little children, and his indignation at their wrongs, will surely feel that the death of Ernest Hart has left a gap which will not easily be filled.

